# "Tricuspid valved patch" repair with a polytetrafluoroethylene valved conduit for right ventricular outflow reconstruction

**DOI:** 10.1016/j.atssr.2025.03.010

**Published:** 2025-03-26

**Authors:** Yusuke Yamamoto, Hajime Sakurai, Takafumi Terada, Masato Mutsuga

**Affiliations:** 1 Department of Cardiac Surgery, Nagoya University Graduate School of Medicine, Nagoya, Japan

## Abstract

As an alternative to bioprosthetic pulmonary valve replacement for patients with late pulmonary insufficiency of repaired tetralogy of Fallot, we developed a novel technique using a handmade polytetrafluoroethylene tricuspid valved conduit, wherein the 2 posterior sinuses of the valve were resected and interdigitated with the native tissue of the pulmonary sinuses, followed by augmentation of the pulmonary trunk with the anterior wall of the conduit. Reliable valvular function of the tricuspid valve and the favorable biocompatibility of polytetrafluoroethylene material along with the growth potential of the repaired right ventricular outflow tract suggest that it may be an optimal alternative especially for young patients.

Late pulmonary insufficiency is common in patients with repaired tetralogy of Fallot, especially in those who have undergone transannular patch repair. Pulmonary valve replacement (PVR) with a bioprosthetic valve is a common option in these patients, although suboptimal durability of the xenograft material is of great concern in younger patients. As an alternative to bioprosthetic PVR, we developed a novel technique using a handmade polytetrafluoroethylene (PTFE) tricuspid valved conduit, wherein the 2 posterior sinuses of the valve were resected and interdigitated with the native tissue of the pulmonary sinuses, followed by augmentation of the pulmonary trunk with the anterior wall of the conduit. Favorable biocompatibility of PTFE material and competent valvular function with the tricuspid configuration might contribute to the long-term durability of the repaired right ventricular outflow tract (RVOT) in younger patients. Herein, we describe a 7-year-old girl with severe pulmonary insufficiency who was successfully treated with this technique. She had undergone total correction of a double outlet right ventricle with a transannular patch during infancy, and surgical reintervention was indicated for the aggravation of her pulmonary insufficiency.

## Technique

**Figure 1:**
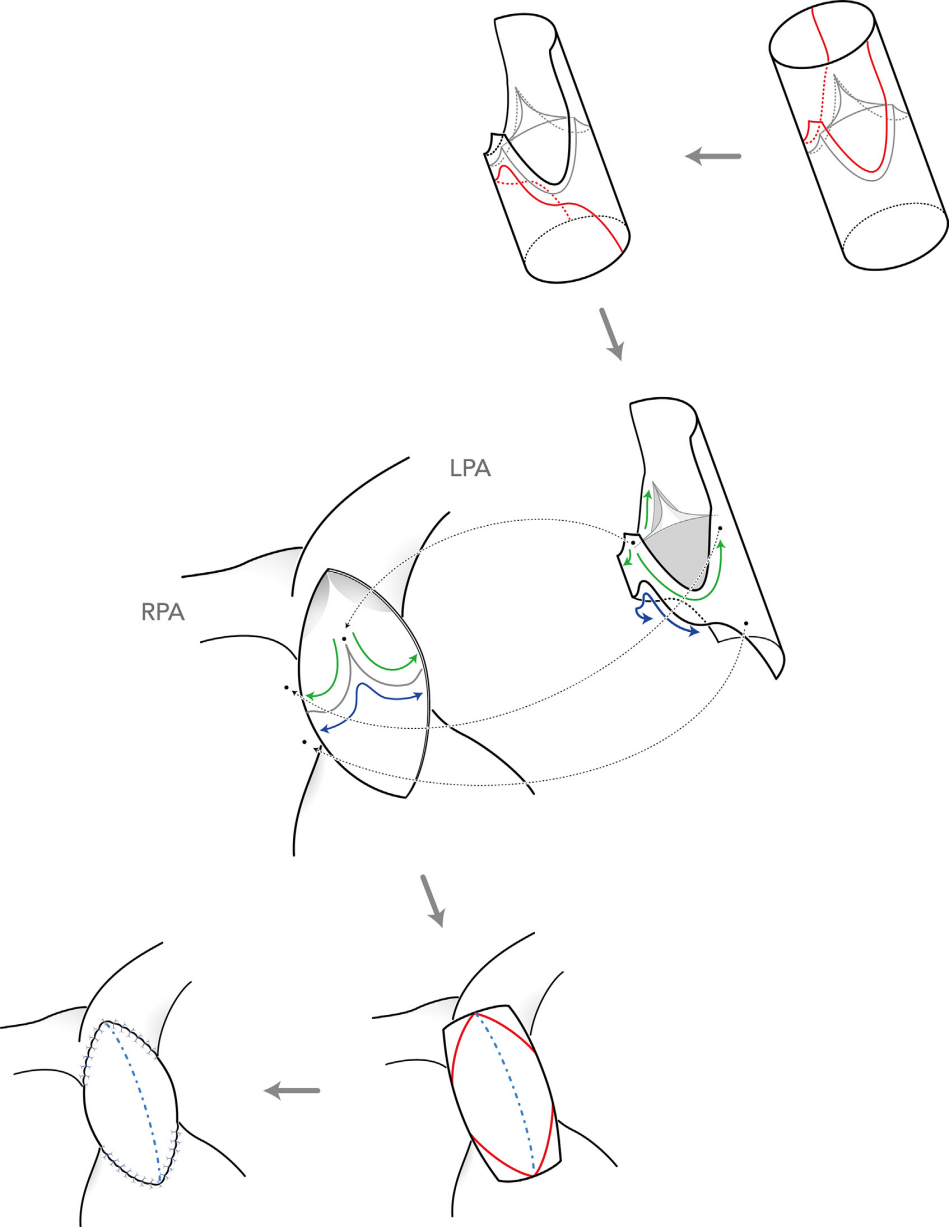
Stepwise illustration of the tricuspid valved patch technique. Incision line of the conduit is indicated by the red line. First, the 2 posterior sinuses of the conduit were resected, corresponding to the shape of the native sinuses of the pulmonary root, and the posterior interleaflet triangle was also resected from the proximal stump of the conduit. Then, the posterior two-thirds of the distal stump was sutured to the annulus of the pulmonary valve (green line); the proximal stump was sutured to the posterior wall of the right ventricular outflow tract with an upwardly convex suture line below the pulmonary annulus (blue line). Finally, after resection of the excessive part of the conduit wall, the incised right ventricular outflow tract was augmented with the remaining anterior wall of the conduit in the same way as in conventional transannular patch repair. (LPA, left pulmonary artery; RPA, right pulmonary artery.)

Before the operation, a tricuspid valved conduit was prepared in the operating room using a PTFE tubular graft, with a leaflet cut out from a 0.1-mm-thick PTFE membrane in a fan-shaped design originally developed in Kyoto.^1^ Through median sternotomy, cardiopulmonary bypass was established with aorta-bicaval cannulation (Video 1). After antegrade cardioplegic arrest, the previous transannular patch was completely removed. After inspection of the inside of the pulmonary trunk, the 2 posterior sinuses of the conduit were resected, leaving a margin of 3 mm in width from the suture line of the PTFE leaflet, and the posterior interleaflet triangle was also resected from the proximal stump of the conduit. Then, the posterior two-thirds of the distal stump was sutured to the annulus of the pulmonary valve; the proximal stump was sutured to the posterior wall of the RVOT using an upwardly convex suture line below the pulmonary annulus, taking care not to injure the left coronary artery behind by deep stitches (Figure 1). Finally, the incised RVOT was augmented with the remaining anterior wall of the conduit, in the same way as in a conventional transannular patch repair. Good valvular function with preserved leaflet motion was confirmed by postoperative echocardiography (Video 2).

## Comment

**Figure 2:**
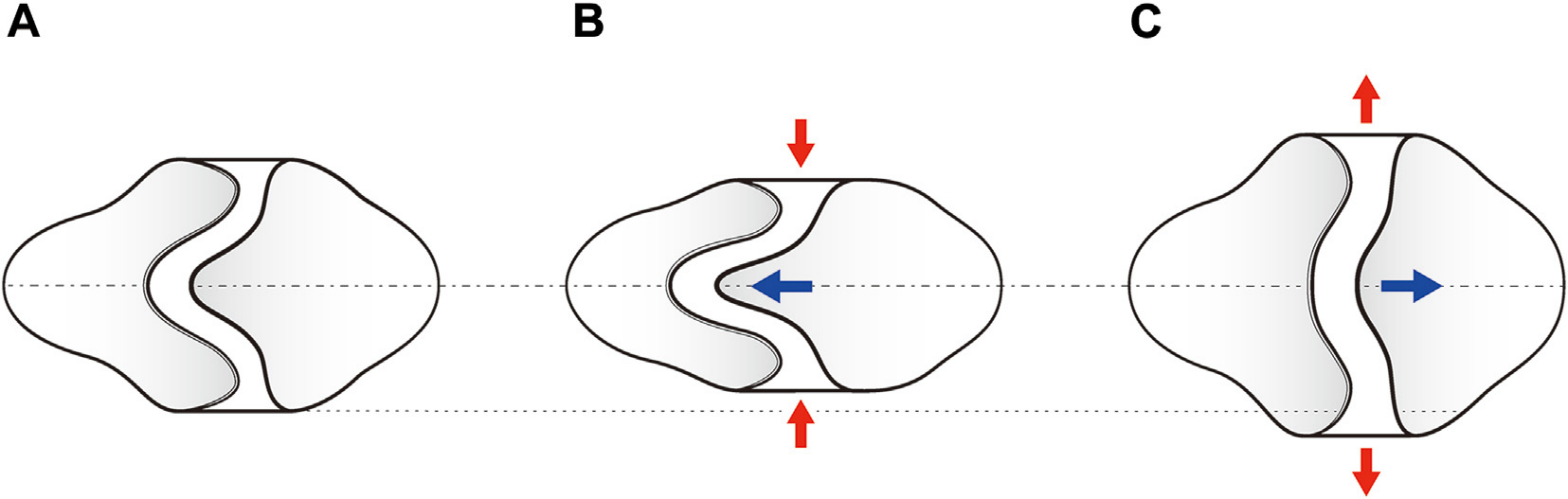
Schematic view of the growth potential of the implanted tricuspid valved patch. (A) Posterior aspect of the tricuspid valved patch in its original shape. The skeletal posterior annulus of the polytetrafluoroethylene valve is achieved by removing 2 sinuses and the interleaflet triangle between them. (B) The longitudinal traction of the posterior commissure reduces the actual diameter of the valvular annulus. This folding effect may allow implantation of a larger valve compared with the conventional Rastelli procedure. (C) Expected annular enlargement along with the somatic growth of the patient.

Late RVOT reintervention remains a significant problem after surgical repair of tetralogy of Fallot or its related diseases, occurring in about 30% of cases, especially in those who have undergone transannular patch repair.^2^ Although bioprosthetic PVR is the most common therapeutic strategy for this condition, clinical outcomes in younger patients are unfavorable, possibly because of the active immune response in this population.^3^ In contrast, satisfactory long-term outcomes after RVOT repair with the PTFE tricuspid valved conduit in children and adolescents have been reported.^4^ The low antigenicity of PTFE material and hemodynamic advantage of the bulging sinus might explain this superiority of the PTFE valved conduit. Nevertheless, because of technical difficulties in molding sinuses onto the PTFE graft, conduits with bulging sinuses are available only at limited institutions, whereas tubular grafts are the only option at most institutions in the whole world. To address the hemodynamic disadvantage of the valved conduit with tubular PTFE graft, we developed a novel technique wherein the 2 resected posterior sinuses of the conduit are remodeled using autologous pulmonary sinuses, which are expected to reproduce the analogous hemodynamic properties of the pulmonary root. In addition, while achieving good valvular function comparable to the Rastelli procedure, this technique is simple and less invasive, similar to transannular patch repair. Furthermore, the longitudinal foldability of the skeletal posterior annulus may allow implantation of a larger valve than that used in the Rastelli procedure; future annular enlargement may be provided by extension of the folded annulus along with somatic growth of the patient (Figure 2).

In summary, our novel tricuspid valved patch technique is feasible and affordable using only commercially available materials. The growth potential of the repaired RVOT along with the favorable biocompatibility of PTFE material suggests that it may be an optimal alternative especially for young patients. Both further follow-up of the long-term outcomes and fluid dynamic studies of the implanted PTFE valve remain subjects for future investigations.
